# The Physiology of the Different Varieties of Paralysis

**Published:** 1856-01

**Authors:** Marshall Hall

**Affiliations:** London


### THE PHYSIOLOGY OF THE DIFFERENT VARIETIES OF PARALYSIS.

BY MARSHALL HALL, M. D., F. R. S., LONDON.

(Read before the Institute of France, Academy of Sciences.)

As there are two principal nervous centres, the brain and the spinal cord, so there are two great classes of paralysis, according as the influence of the brain or spinal system is intercepted or annihilated.

I denominate cases belonging to the first class, in which the palsied parts are deprived of the influence of the brain, *cerebral paralysis.* Cases in which the influence of the spinal cord is intercepted from my second class, *spinal paralysis.* I do not mean to imply by these terms that there are, in these cases respectively, lesions of the encephalon or spinal cord, but simply that by some disease or injury the influence of these organs is abolished, so far as the muscles of the palsied limbs are concerned. Hemiplegia is ordinarily a cerebral paralysis; but in some cases, a spinal paralysis also; whereas disease limited to a small part of the dorsal segment of the cord produces a cerebral paralysis of the lower extremities; the influence of the portion of spinal cord below the seat of disease continuing to reach the palsied limbs. The destruction of a considerable portion of the spinal cord, or a suspension or annihilation of the functions of the spinal cord produces a spinal paralysis.

A cerebral paralysis, I repeat, is one in which the muscles are deprived of the influence of the brain; a spinal paralysis, one in which the muscles are deprived of the influence of the spinal cord.

Facial hemiplegia is a cerebral paralysis; paralysis of the facial nerve is a spinal paralysis. The distinctive characteristics ot these two classes of palsies are as follows:—

In cerebral paralysis, the influence of the will is alone interrupted. When this paralysis is complete, voluntary movements are abolished. All the functions depending on the medulla oblongata and spinal cord persist. We have, in different cases:

1. Emotional movements;
2. Movements connected with yawning, coughing, etc.;
3. Diastolic movements;
4. Tonic symmetrical contractions of the hands;
5. Comparative increase in the irritability of Haller;
6. Comparative increase in susceptibility to the action of strychnia.

In spinal paralysis, the four species of movements above enumerated are not observed, and the Hallerian irritability is comparatively less.

I return to cases of hemiplegia. In most cases, shortly after the attack, there is somewhat of an amelioration, a partial return of voluntary power; the phenomena I have mentioned are manifested also. In other cases there is no amelioration; the phenomena adverted to are absent or scarcely perceptible. There are no tonic spasms of the hand and arm; the Hallerian irritability is not augmented. It might be said that such cases were exceptions to the rules I have laid down. The truth is, it appears to me, that, in such instances, the shock of the attack has been sufficient to destroy, so to speak, the nervous power of the spinal system. Thus, when we divide the spinal marrow of the frog from the brain by an incision, we suspend nervous power, so as to abolish diastolic movements. A yet more violent shock, as a stroke of lightning, would annihilate it altogether.

These phenomena are objects of pure observation, excepting that relating to irritability. To test this function of the muscular fibre, I have experimented on various occasions, with the aid of galvanism, and repeated my experiments with every precaution.

I made use of a simple galvanic current, produced by a Cruikshank machine. I placed a palsied and a sound hand, for example, in the same basin of pure water, and the feet in another, and carefully observed which was affected by the slightest degree of galvanism. I found that in cerebral paralysis, the palsied limb is most susceptible of galvanic excitation; whereas in spinal paralysis, the palsied limb is less susceptible than the sound one.

I deduce from these experiments many conclusions of interest both to the physiologist and the physician.

1. That the brain, by its acts of volition, tends to exhaust muscular irritability.

2. That the spinal marrow, on the contrary, is the source of this irritability.

3. That galvanism will serve to diagnosticate between cerebral paralysis and spinal paralysis.

The phenomena I have already enumerated: yawning, the effects of emotion, diastolic movements, symmetrical tonic spasms, the effects of strychnia, etc.

Besides cerebral and spinal paralysis, there are nervous affections connected with the medulla oblongata and pneumogastric nerves, which I propose to discuss on a future occasion, as well as the diseases of the ganglionic system.

Lastly, to complete our enumeration of paralysis, there remain several varieties of palsy that are exceedingly obscure; paralysis *cum agitatione;* paralysis *e plumbo*, *e rheumatismo*, *ex hysteria*,

*e dentitione*, etc. Much labor is requisite before we can form clear ideas on these diseases. Emotions, spinal irritation, the action of poisons, the influence of pain, the effect of shock; what a field for study!

